# Paratesticular fibrous pseudotumor in young males presenting with histological features of IgG4-related disease: two case reports

**DOI:** 10.1186/1752-1947-7-225

**Published:** 2013-09-11

**Authors:** Klaus-Peter Dieckmann, Werner Jan Struss, Ulrich Frey, Martina Nahler-Wildenhain

**Affiliations:** 1 Klinik für Urologie, Albertinen-Krankenhaus Hamburg, Suentelstrasse 11a, 22457 Hamburg, Germany; 2 Abteilung für Radiologie, Albertinen-Krankenhaus Hamburg, Suentelstrasse 11a, 22457 Hamburg, Germany; 3 Institut für Pathologie, Albertinen-Krankenhaus Hamburg, Fangdieckstrasse 75a, 22547 Hamburg, Germany

**Keywords:** Paratesticular pseudotumor, IgG4-related disease, Scrotal magnetic resonance imaging, Scrotal ultrasound, Testis-sparing surgery

## Abstract

**Introduction:**

Paratesticular fibrous pseudotumors represent benign new growths confined to intrascrotal structures. Both pathogenesis and clinical management are little understood due to the rarity of the lesion, with less than 200 cases reported to date. Recently, paratesticular fibrous pseudotumors have been postulated to be immunoglobulin G4-related, pathogenetically. Here we report two cases of patients with paratesticular fibrous pseudotumor to highlight the clinical features of this rare disease and we report the immunohistochemical examinations to support the theory of paratesticular fibrous pseudotumor being an immunoglobulin G4-related disease.

**Case presentations:**

A 28-year-old white man presented with a painless intrascrotal mass. After a clinical examination, a malignant growth was suspected. His ultrasound results revealed a well-demarcated hypoechoic lesion of 1.5cm in diameter at the spermatic cord. Our patient underwent local excision. His follow-up has been uneventful for 12 years. The second case was an 18-year-old white man who presented with a painless scrotal mass suspicious of testicular tumor. A magnetic resonance imaging scan revealed a 3cm mass at the spermatic cord with very low signal density on T2-weighted imaging and a low and inhomogeneous uptake of gadolinium contrast agent on T1-weighted, fat-suppressed imaging. Following local excision, our patient has been well for 18 months.

On histological examination, both of the lesions consisted of collagen-rich hyalinized fibrotic tissue with storiform features. There were lymphofollicular infiltrates and, sporadically, also venulitis. The immunoglobulin G4 staining (in case 2) showed an infiltrate of 10 to 15 positive cells per high-power field on average, corresponding to a proportion of 40% in evaluable hot spots. The two patients with paratesticular fibrous pseudotumor presented within a time span of 15 years. During that time, 400 patients with testicular germ cell tumors had been treated in our institution.

**Conclusions:**

The specific histological features documented in our case lend support to the theory of paratesticular fibrous pseudotumor being an immunoglobulin G4-related sclerosing disorder. Paratesticular fibrous pseudotumors usually occur in young adulthood. Clinically, paratesticular fibrous pseudotumor can mimic testicular malignancy. Ultrasonographic findings are largely unspecific, however, scrotal magnetic resonance imaging may aid in discriminating the lesion from malignant tumors. Local excision, whenever technically feasible, is the preferred treatment of paratesticular fibrous pseudotumor.

## Introduction

Fibrous pseudotumors represent a clinicopathological challenge because of their unknown origin, and their widely variable topographical as well as morphological features. A fibrous pseudotumor confined to paratesticular structures was first described by Balloch in 1904 [1]. Numerous cases have been reported since then, however, the understanding of this lesion had been caught up by its rarity, confusion with similar but different entities, and by the perplexity of the terminology [2]. Only 6% of paratesticular new growths represent fibrous pseudotumors [3]. In 1973, Mostofi and Price reviewed the cases of paratesticular fibrous pseudotumors (PFP) submitted to the Armed Forces Institute of Pathology [4]. They outlined that PFPs may occur as a single or as disseminated nodules, and rarely as confluent testis-encasing masses. Microscopically, the nodule consists of dense, almost acellular, hyalinized collagen.

Based on the contention that these growths were of reactive rather than neoplastic origin, Mostofi and Price explicitly supported the term ‘fibrous pseudotumors’ [4], which has been adopted by most standard pathology textbooks and guidelines. Accordingly, due to its benign nature, the entity is not listed in the World Health Organization classification of tumors. Recently, PFPs have been suspected to represent an immunoglobulin G4 (IgG4)-mediated disease [5]. Yet so far, no further data have been provided to support this appealing theory.

Clinical management of PFPs is largely unresolved. Misapprehension does occur in the majority of cases mainly because these intrascrotal masses may mimic testicular neoplasms [6]. Due to their rarity, modern imaging techniques, particularly scrotal magnetic resonance imaging (MRI), have only been sporadically studied in these cases [7-9]. Thus, we should like to document two cases of PFP to increase awareness and knowledge of this rare intrascrotal disorder.

## Case presentations

The first case was a 28-year-old white patient who presented with a right-sided painless scrotal mass that had been slowly growing for four years. His medical history did not involve any predisposing conditions. On clinical examination, a hazelnut-like, hard and mobile nodule was detected by palpation in the right-sided paratesticular region. An ultrasound scan revealed a 1.5 × 1.5cm clearly demarcated hypoechoic lesion at the spermatic cord cranial to the testicle*.* Surgical exposure by trans-scrotal incision revealed a solid, well-delineated nodule at the spermatic cord distinct from the testis and the epididymis. Local excision was easily accomplished. His postoperative recovery was uneventful. After 12 years of follow-up neither recurrence of intrascrotal disorders nor any other serious diseases have been noted.

**Figure 1:**
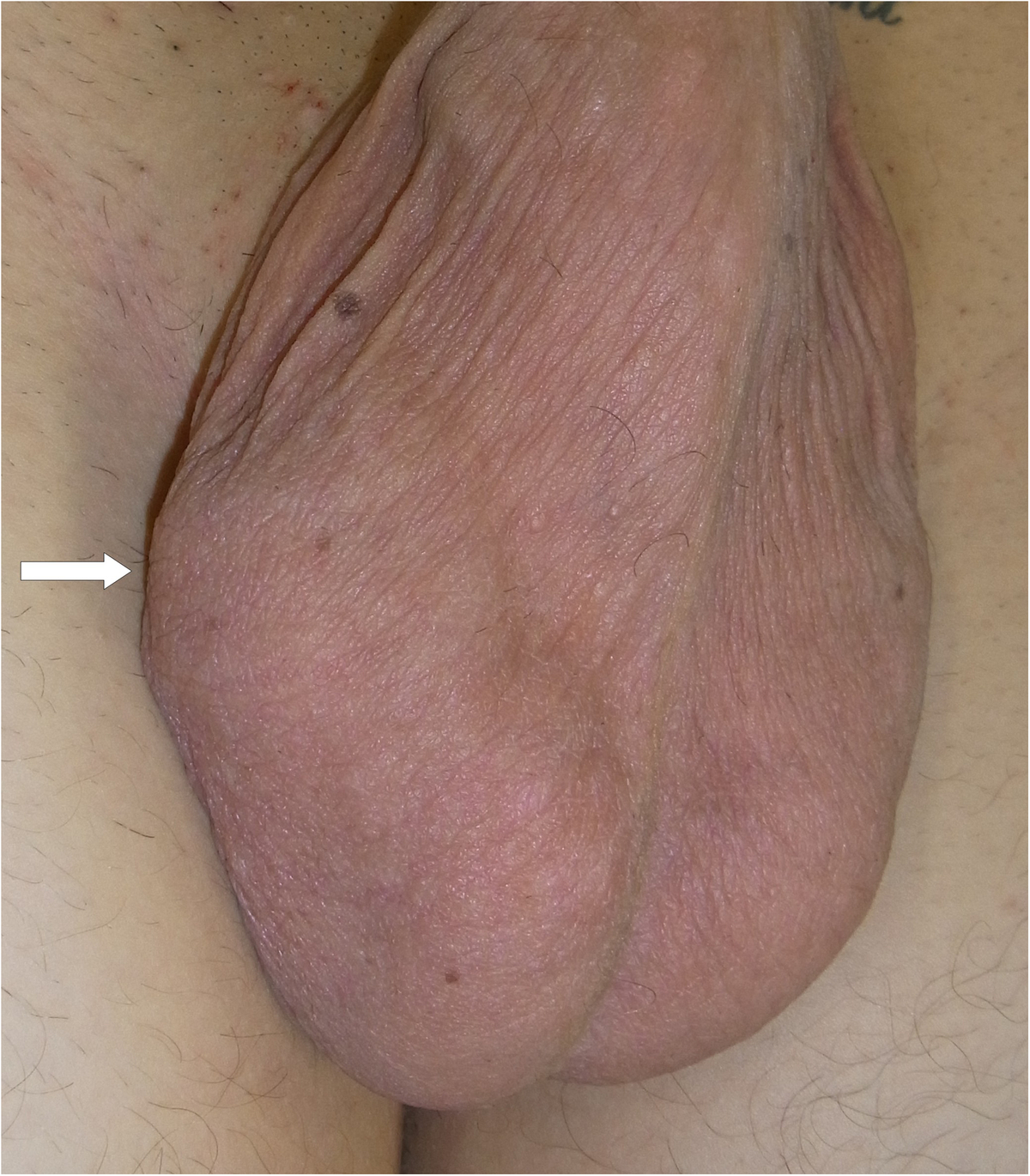
Clinical view: note the intrascrotal mass located cephalad to the right testicle (arrow).

The second case was a 19-year-old white man who presented with a right-sided painless scrotal mass that had appeared six months previously. His medical history involved a tonsillectomy but no specific events predisposing to scrotal diseases. Our patient was in excellent general condition. A prune-sized solid mass was palpable in the paratesticular region (Figure 1).

Grey scale ultrasonography with a 10Mhz transducer revealed a well-demarcated hypoechoic lesion with a homogeneous echo pattern of 3 × 3cm size cephalad to the testicle.

**Figure 2:**
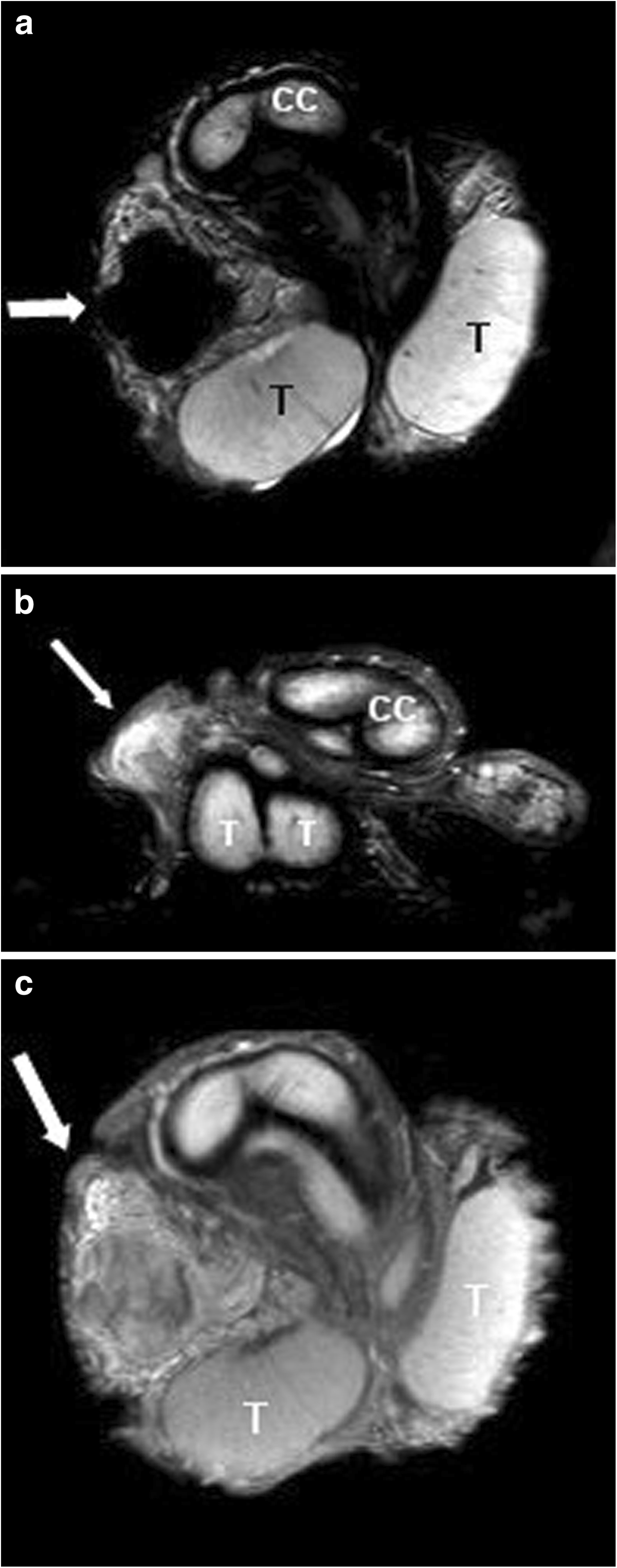
**Magnetic resonance imaging (1.5 Tesla) with a surface coil. (a)** Polycyclic mass (arrow) cephalad to the right testicle with no signal intensity on T2-weighted imaging. The mass is clearly detached from the testicle (coronary section). **(b)** The intrascrotal mass (arrow) shows inhomogeneous signal intensity on T1-weighted imaging (coronary section). **(c)** The mass (arrow) shows only low and inhomogeneous uptake of gadolinium contrast agent (coronary section). CC, corpus cavernosum; T, testicle.

An MRI scan, using a 1.5 Tesla machine with the application of a surface coil, confirmed a well-circumscribed polycyclic mass of 3.5cm in diameter confined to the spermatic cord, clearly detached from the testicle and epididymis. On T2-weighted imaging the lesion revealed very low signal density (Figure 2a) whereas T1-weighted imaging disclosed intermediate signal density of the lesion, mirroring the typical findings in T1-weighted skeletal muscle imaging (Figure 2b). Contrast-enhanced imaging (T1-weighted and fat-suppressed) revealed a low and inhomogeneous uptake of gadolinium (Figure 2c).

Laboratory tests did not disclose any anomalies.

**Figure 3:**
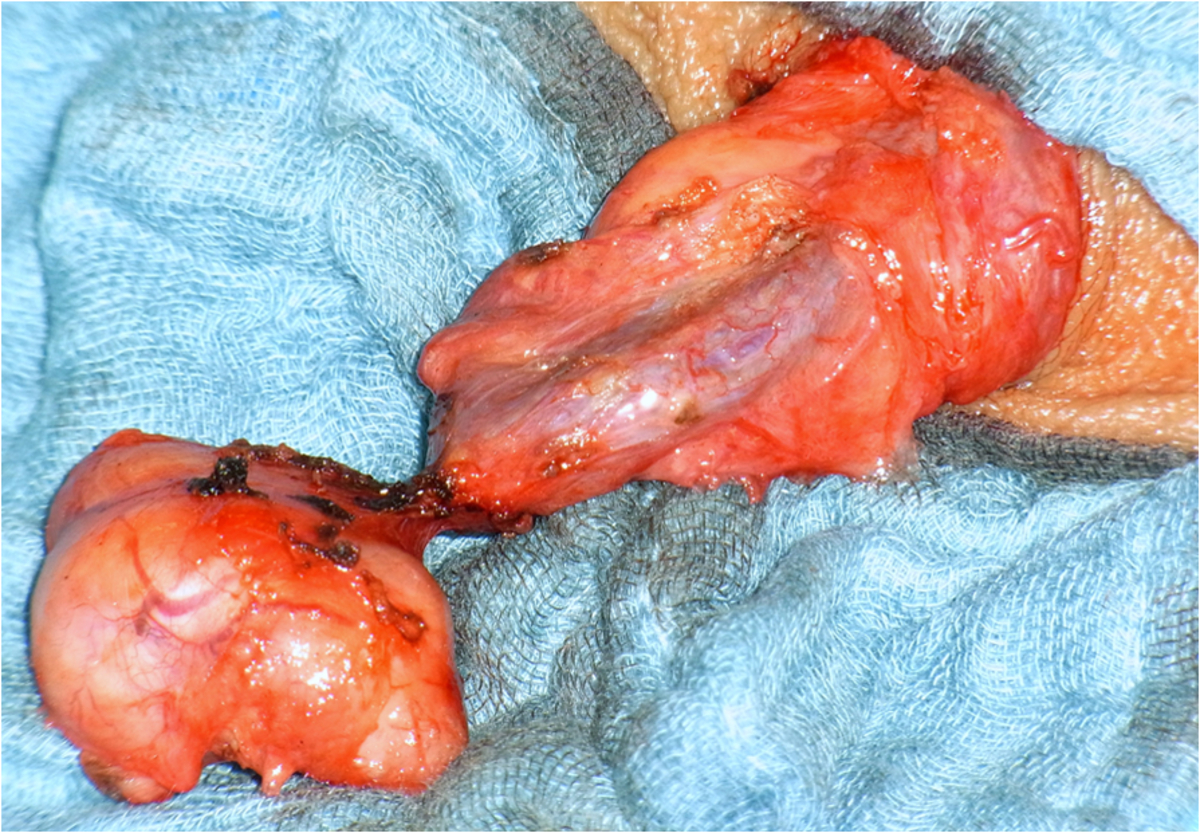
Intraoperative view: note the pedunculated mass originating from the spermatic cord.

Upon surgical exposure, a firm pedunculated mass originating from the spermatic cord was found. The lesion was completely excised without threat to the remaining scrotal content (Figure 3). His postoperative recovery was uneventful. After 12 months of follow-up, our patient is well without any complaints regarding his scrotal content.

**Figure 4:**
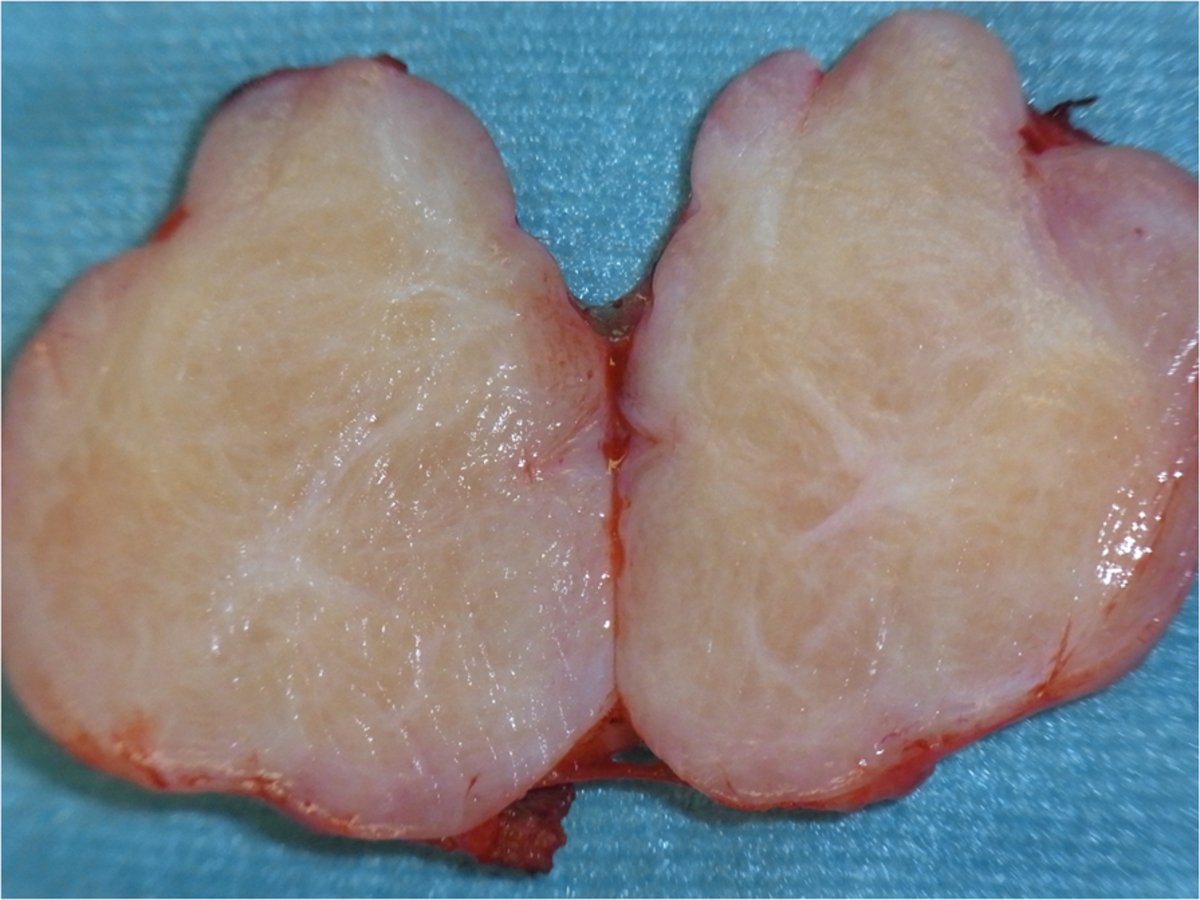
Surgical specimen: yellowish-white homogeneous cut surface and well-demarcated margins of the lesion.

In both cases, the excised masses presented with identical morphological features. Grossly, they showed a yellowish-white cut surface, were stony hard in consistency and were 1.5cm and 3.5cm in diameter, respectively (Figure 4).

**Figure 5:**
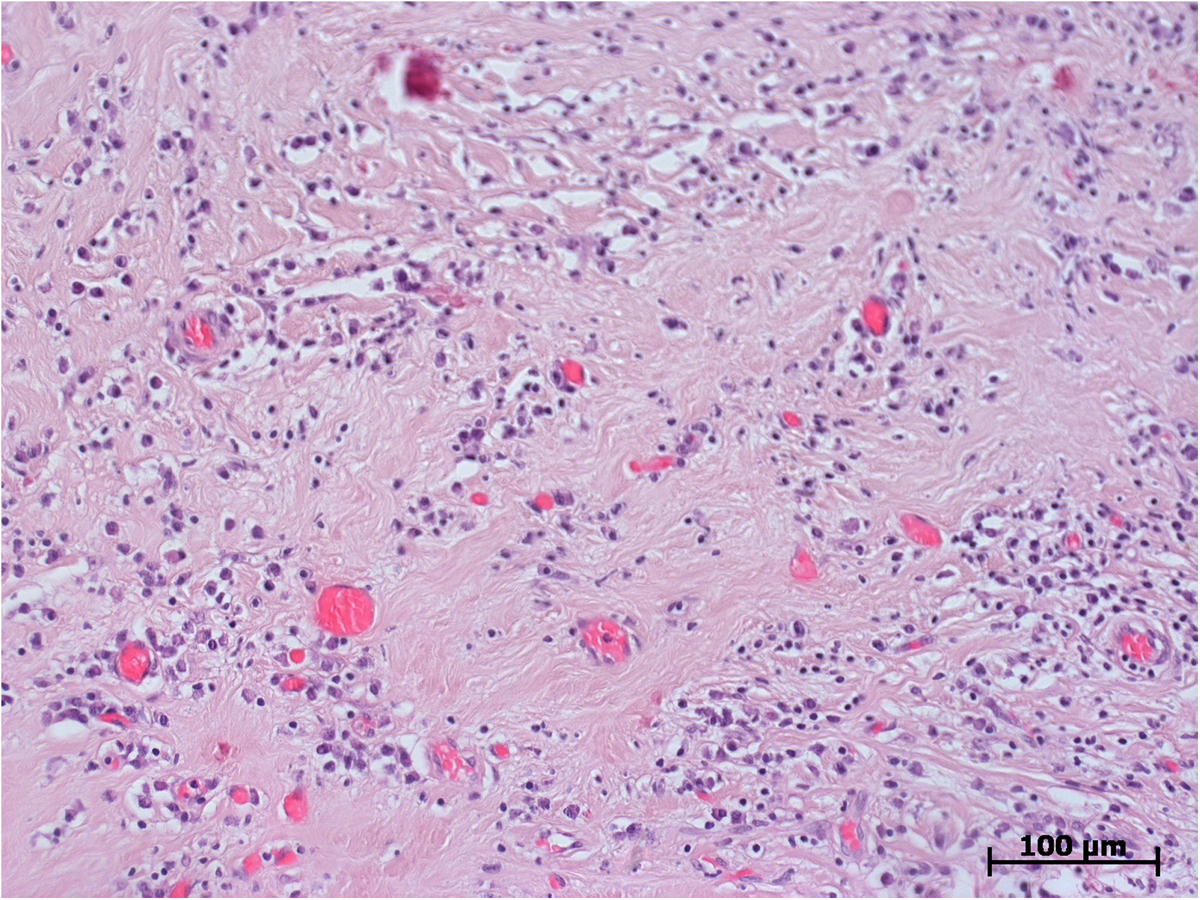
**Histological section: dense fibrotic tissue with interspersed lymphatic cells and multiple tiny calcifications (red).** Hematoxylin and eosin staining, original ×200.

**Figure 6:**
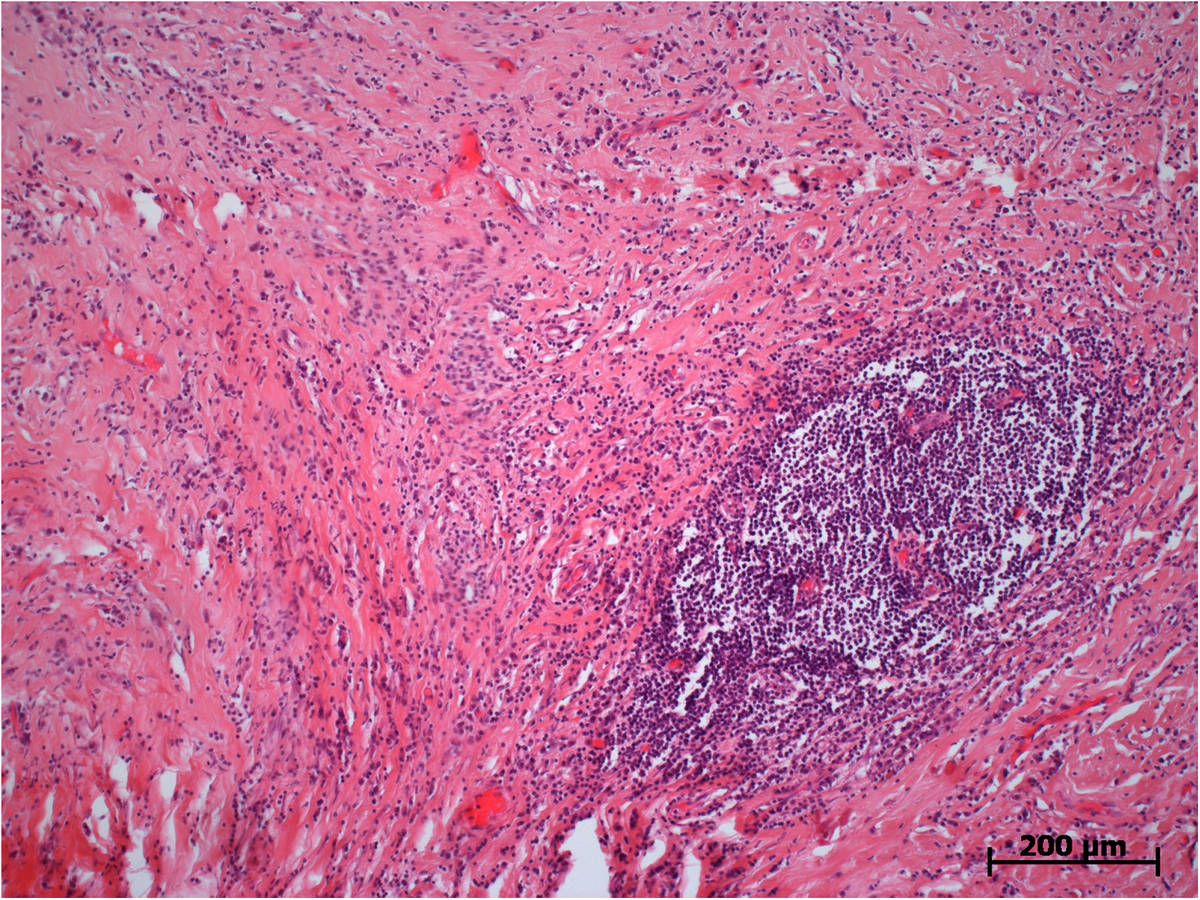
**Histological section: dense collagen-rich hyalinized fibrotic tissue with a lymphofollicular infiltrate.** Hematoxylin and eosin staining, original ×100.

**Figure 7:**
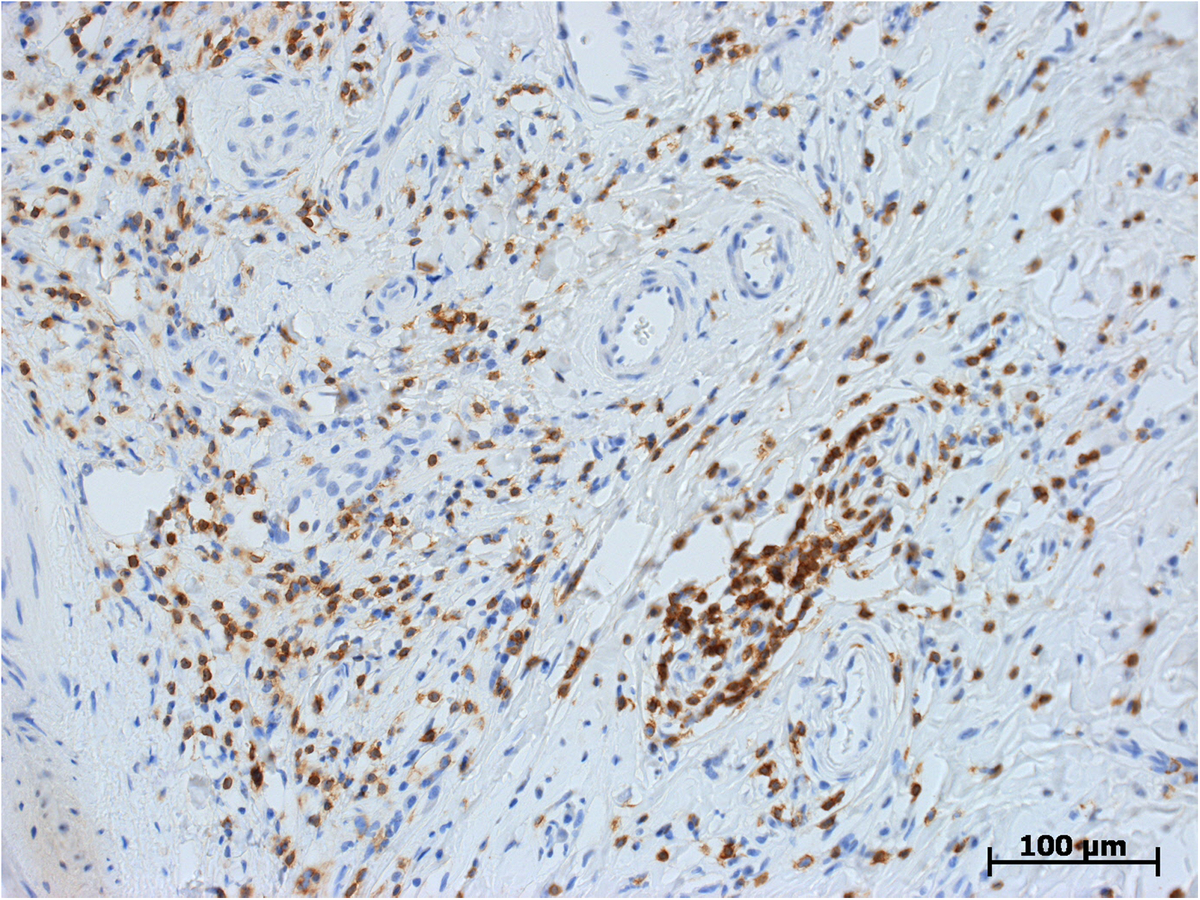
**Histological section: immunohistochemical staining of CD3 showing a dense infiltrate of lymphocytes.** CD3, original ×200.

**Figure 8:**
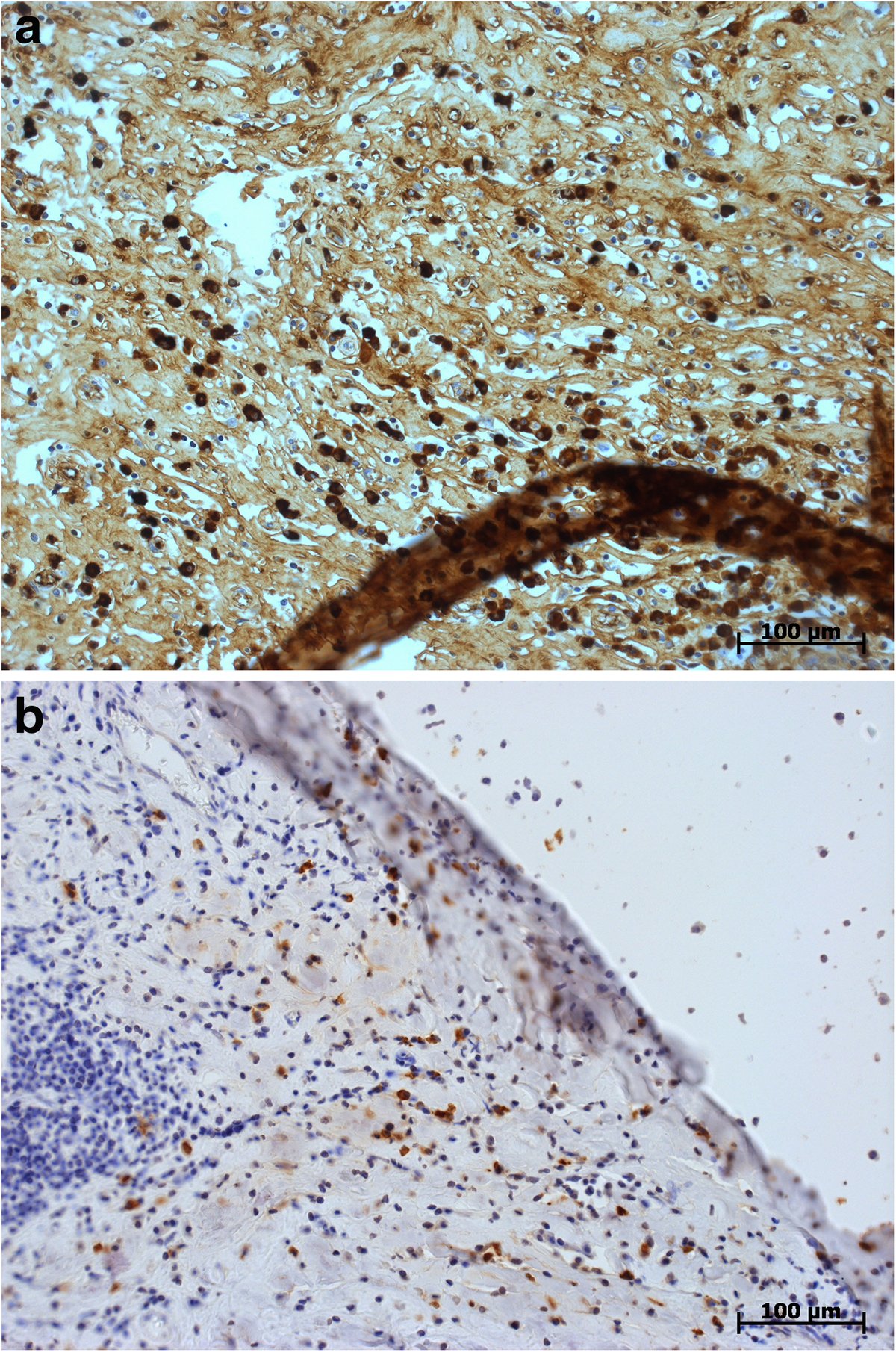
**Histological section: immunohistochemical staining of immunoglobulin G. (a)** Note the dense infiltrate of plasma cells (brownish-stained). (**b)** Note the large proportion of immunoglobulin G4-positive plasma cells. Immunoglobulin G4, original ×200.

**Figure 9:**
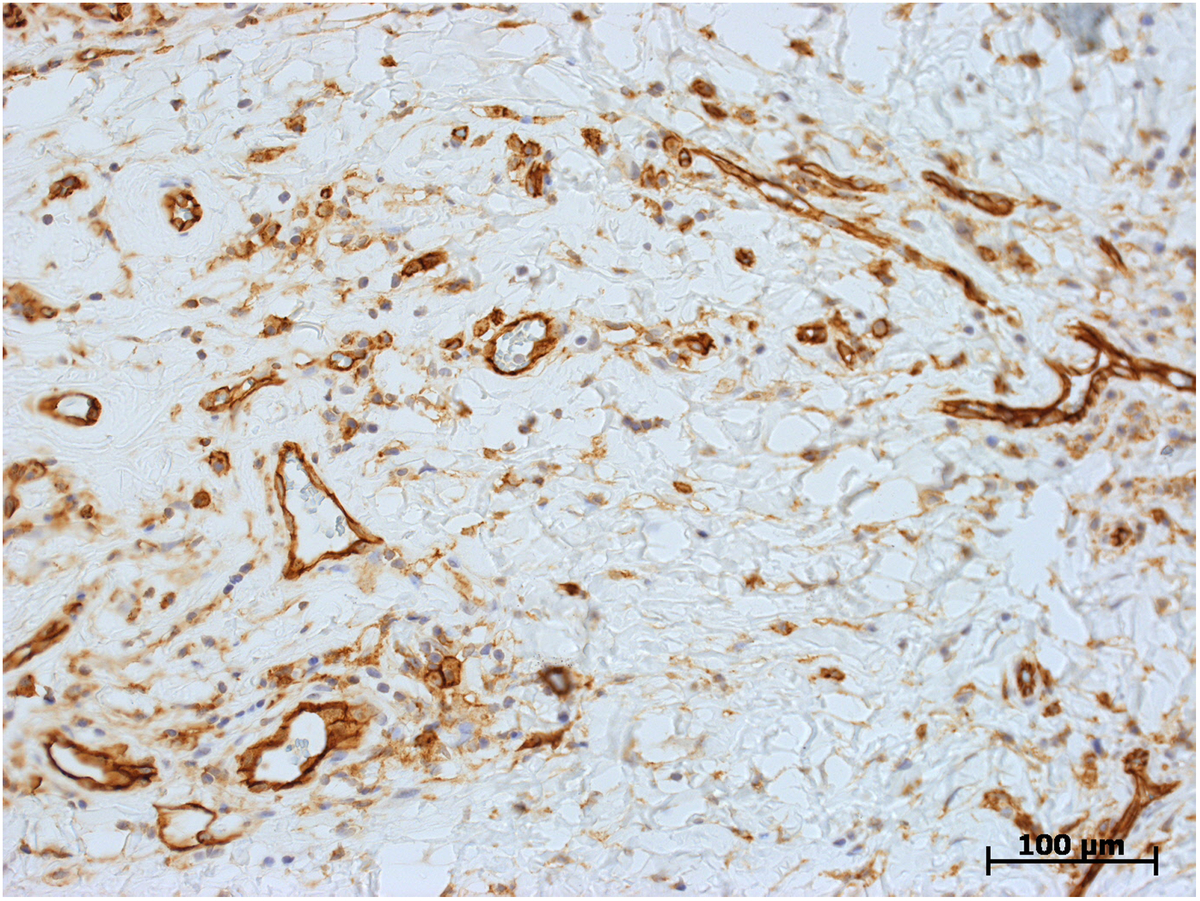
**Histological section: immunohistochemical staining of CD31 showing a high number of blood vessel cross sections.** CD31, original ×200.

Microscopically, both specimens were composed of dense, collagen-rich, hyalinized, partly storiform fibrotic tissue with tiny calcifications (Figure 5) and a low cell density of interspersed spindle-like cells and lymphofollicular infiltrates (Figure 6). Immunohistochemistry, which could only be performed on case 2, demonstrated positive staining results for actin, D2-40, and phosphoglucomutase 1 (PGM1). Staining results were negative for cytokeratin, calretinin, caldesmon and S-100 as well as for germ cell tumor markers Oct 3/4 and placental alkaline phosphatase (PLAP). Of note, there were diffuse to patchy infiltrates predominantly consisting of CD3+ T lymphocytes (Figure 7) and IgG-positive plasma cells (Figure 8a). The IgG4 staining result disclosed 10 to 15 positive cells on average per high-power field (Figure 8b), corresponding to a proportion of approximately 40% of IgG-positive cells in evaluable hot spots. Rather unexpectedly, there was also considerable vascularization detected by the staining of CD31 and CD34 (Figure 9). Venulitis was found sporadically and only in small venules. The proliferation rate was approximately 5% as demonstrated by molecular immunology Borstel-1 (MIB-1) staining.

## Discussion

The overall incidence of PFP is exceptionally rare with approximately 200 cases reported to date. Clearly, underreporting must be considered as well as misrecognizing the lesion due to confusing PFP with other benign scrotal entities [6]. We experienced two patients with PFP within a time span of 15 years. Roughly 400 patients with testicular germ cell tumors (GCTs) presented during the same period as well as six cases with adenomatoid tumor and three other rare paratesticular tumor-like lesions (one sarcoidosis, two malacoplakia). Thus, the relative incidence of PFP in relation to GCT is 1:200, and PFP is the second most frequent benign lesion of the paratesticular region following adenomatoid tumors. So, our experience is in line with reported data [3,4].

In a recent review, Miyamoto *et al.* (2010) reported a mean age at presentation of 42 years [10]. Both of the patients presented herein were considerably younger (19 and 28 years, respectively). In fact, isolated PFP cases have been encountered in children, too [11]. It thus appears that PFPs have their peak incidence in young adulthood but sporadically they may occur in adolescence. Probably, this age predisposition is one of the reasons why these scrotal masses may be clinically mishandled as testicular neoplasms.

According to previous reports, PFPs would more commonly arise on the left side [11]. Noteworthy, both of our cases were localized on the right side. Thus, evidence pointing to the preponderance of one particular side is probably rather scant.

With respect to localization, PFPs are observed on the tunica vaginalis, the epididymis, and the tunica albuginea in 76%, 10%, and 14% of cases, respectively [4]. So, localization of a PFP distant from the tunica vaginalis at the spermatic cord, as found in our second patient, is rather unusual.

Clinically, both of our cases presented as painless intrascrotal nodules. Their history was remarkably long, comprising of four years in the first of our cases, however, similar findings have been reported previously [11].

Suspicion of testicular malignancy had prompted admission to hospital in both of our cases. Fortunately, preoperative scrotal imaging afforded excluding of this diagnosis and pursuing a testis-sparing surgical approach in both cases. The typical ultrasonographic features of PFP involve sharp margins of the lesion, and localization of the lesion outside of the testicular parenchyma. Usually, the sonographic pattern is homogeneously hypoechoic [12]. MRI of PFP has been reported in only four cases to date [7-9]. Typical findings involve a very low signal density on T2-weighted imaging, intermediate signal density on T1-weighted scanning and a low and inhomogeneous uptake of gadolinium contrast agent.

Histologically, PFPs present with the typical histomorphological picture of paucicellular hyalinized tissue with bundles of collagen fibers interspersed by spindle cells and plasma cells as well as lymphocytes [6]. Immunohistochemical staining verifies the nonepithelial mesenchymal origin of the growth, with positive results for vimentin and actin and negative results for cytokeratin and desmin, respectively [2]. The proliferation index of 5% appears notably higher than expected in connective tissue but is in accordance with the characteristics of a slowly growing mass. The histopathological findings in our cases are well in line with previous reports. However, the rather unspecific features are obviously the reason for the perplexity of terms used for PFP as well as the reason for confusing PFP with other entities.

Notwithstanding, a probably more specific observation is the significantly elevated IgG4 cell count that was first reported by Bösmüller *et al.*[5] and now confirmed here. For establishing the diagnosis of an IgG4-related sclerosing disease, four morphologic features are required: (1) a proportion of (at least) 40% IgG4-positive cells among IgG-producing plasma cells, (2) a predominantly T-lymphocytic, often lymphofollicular, infiltrate, (3) storiform fibrosis and (4) venulitis [13]. If three out of four criteria are met, the disease is highly probable. Yet, the extent of fibrosis and the density of the cellular infiltrate may vary considerably according to the involved organ. In the present cases, there is evidence of all of the four characteristic features, although venulitis is only sporadically observed in small venules. Immunohistochemistry demonstrates a larger lymphoplasmacytic infiltrate than expected from hematoxylin and eosin (H & E) slides, although plasma cells are easily identified on conventional stains. In-depth workup of the specimens was considerably hampered by the calcification of the tissue in the present cases. Nonetheless, the total histological results obtained in our cases lend support to the theory of PFPs being an IgG4-related sclerosing disorder [5,13,14]. The goal of the present report is to raise awareness of PFP and its presumed pathogenesis, to thus allow future cases to receive targeted histological processing after gentle decalcification. In addition, IgG4 elevation in serum should be measured to support the diagnosis. Unfortunately, immunoglobulin serum profiles had not been taken in our patients because the pathogenetic relevance of this test had not been considered during the clinical management of the two patients.

With respect to the treatment of PFP, conservative surgery, that is local excision of the mass, is sufficient [12]. Our two patients were treated accordingly and the therapeutic results are in line with previous reports. As these growths are commonly misapprehended as malignant neoplasms, clinical management clearly rests on appropriate preoperative imaging technology. Scrotal ultrasound is the mainstay of diagnostics and can direct preoperative decision-making. In cases of doubt, scrotal MRI is a powerful tool to assess the mass even more precisely [7-9]. Thus, unwarranted orchiectomy can be avoided. If the dignity of the mass is still uncertain during surgical exposure, intraoperative frozen section examination can help to prevent unnecessary radical surgery [15].

## Conclusions

Paratesticular fibrous pseudotumors represent a truly rare entity with a peak incidence in young adulthood. There is growing evidence that these nonmalignant growths belong to the group of IgG4-related sclerosing diseases. Clinically, PFPs may be confused with malignant testicular tumors. Ultrasonography and particularly scrotal MRI provide appropriate preoperative assessment of the lesion. Testis-sparing excision of the nodule should always be attempted.

## Consent

Written informed consent was obtained from the two patients for publication of this case report and any accompanying images. Copies of the written consent forms are available for review by the Editor-in-Chief of this journal.

## Competing interests

The authors declare that they have no competing interests.

## Authors’ contributions

KPD conceived the study, analyzed and interpreted the clinical parameters of the patients in light of the available literature, and wrote most parts of the manuscript. WJS performed the surgical procedures, assisted in the clinical management of the patients, took responsibility for obtaining informed consent for publication, made substantial contributions to the acquisition of clinical data, and was an important participant in writing and editing the manuscript. UF performed and analyzed the imaging procedures performed on the patients and was a major participant in writing the manuscript. MNW performed the histological examination of the surgical specimens, and wrote the pathohistological sections of the manuscript. All authors read and approved the final manuscript.

## Authors’ information

KPD is a consultant urologist and has been working in clinical urology for 30 years. He has a particular interest and expertise in scrotal diseases. WJS is a trainee physician in urology. UF is a senior radiologist with a particular interest and expertise in MR imaging. MNW is a staff pathologist dedicated to surgical pathology.
